# Multi-Locus Genome-Wide Association Studies to Characterize Fusarium Head Blight (FHB) Resistance in Hard Winter Wheat

**DOI:** 10.3389/fpls.2022.946700

**Published:** 2022-07-25

**Authors:** Jinfeng Zhang, Harsimardeep S. Gill, Jyotirmoy Halder, Navreet K. Brar, Shaukat Ali, Amy Bernardo, Paul St. Amand, Guihua Bai, Brent Turnipseed, Sunish K. Sehgal

**Affiliations:** ^1^Department of Agronomy, Horticulture and Plant Science, South Dakota State University, Brookings, SD, United States; ^2^USDA-ARS, Hard Winter Wheat Genetics Research Unit, Manhattan, KS, United States

**Keywords:** FHB resistance, GBS, multi-locus GWAS, hard winter wheat, winter wheat breeding

## Abstract

Fusarium head blight (FHB), caused by the fungus *Fusarium graminearum* Schwabe is an important disease of wheat that causes severe yield losses along with serious quality concerns. Incorporating the host resistance from either wild relatives, landraces, or exotic materials remains challenging and has shown limited success. Therefore, a better understanding of the genetic basis of native FHB resistance in hard winter wheat (HWW) and combining it with major quantitative trait loci (QTLs) can facilitate the development of FHB-resistant cultivars. In this study, we evaluated a set of 257 breeding lines from the South Dakota State University (SDSU) breeding program to uncover the genetic basis of native FHB resistance in the US hard winter wheat. We conducted a multi-locus genome-wide association study (ML-GWAS) with 9,321 high-quality single-nucleotide polymorphisms (SNPs). A total of six distinct marker-trait associations (MTAs) were identified for the FHB disease index (DIS) on five different chromosomes including 2A, 2B, 3B, 4B, and 7A. Further, eight MTAs were identified for Fusarium-damaged kernels (FDK) on six chromosomes including 3B, 5A, 6B, 6D, 7A, and 7B. Out of the 14 significant MTAs, 10 were found in the proximity of previously reported regions for FHB resistance in different wheat classes and were validated in HWW, while four MTAs represent likely novel loci for FHB resistance. Accumulation of favorable alleles of reported MTAs resulted in significantly lower mean DIS and FDK score, demonstrating the additive effect of FHB resistance alleles. Candidate gene analysis for two important MTAs identified several genes with putative proteins of interest; however, further investigation of these regions is needed to identify genes conferring FHB resistance. The current study sheds light on the genetic basis of native FHB resistance in the US HWW germplasm and the resistant lines and MTAs identified in this study will be useful resources for FHB resistance breeding *via* marker-assisted selection.

## Introduction

Fusarium head blight (FHB), also known as scab, is one of the most devastating diseases of wheat primarily caused by the fungus *Fusarium graminearum* Schwabe. FHB can cause severe losses in yield due to shriveled grains that lowers the test weight (Gilbert and Tekauz, 2000; Bai and Shaner, 2004). Further, *Fusarium* sp. produces harmful mycotoxins including deoxynivalenol (DON) that can accumulate in the infected grains and poses a serious threat to food and feed safety (Pestka, 2010; Ferrigo et al., 2016). In the US, FHB was first reported by Arthur (1891) in Indiana and since then, FHB has expanded its horizons to all major wheat-growing states in the US. This expansion of FHB is likely due to a suitable climate, increased acreage under no-till cultivation, and adoption of maize-wheat rotations over the last several decades, causing huge economic losses (Nganje et al., 2004; McMullen et al., 2012; Wilson et al., 2017). For instance, the US wheat producers suffered revenue losses worth $850 million due to FHB outbreaks in 2014 alone (Wilson et al., 2017).

Although fungicides are used for FHB prevention and control, the development of FHB-resistant varieties remains the most effective and economical approach to minimize the losses caused by this disease (Bai and Shaner, 2004; Gilbert and Haber, 2013). Genetic resistance to FHB is complex and controlled by multiple quantitative trait loci (QTLs) with small to medium effects. Further resistance expression is also significantly influenced by environmental conditions (Miedaner and Lauber et al., 2001; Buerstmayr et al., 2012). Several types of resistance mechanisms have been proposed, including resistance to the initial infection (Type I), resistance to the spread of infection within the spike (Type II), resistance to accumulation of mycotoxins such as DON (Type III), and resistance against damaged kernels (Type IV; Bai and Shaner, 2004; Gilbert and Haber, 2013), with Type II resistance being more stable and utilized in many wheat breeding programs. Nevertheless, type III and type IV resistance have also received attention in wheat breeding because they are associated with end-use quality and food safety, which is one of the biggest concerns of the growers and food industry (Mesterhazy, 2020). A large number of QTLs have been identified on all 21 wheat chromosomes (Liu et al., 2009; Venske et al., 2019), including seven cataloged FHB genes, *Fhb1* to *Fhb7* (Liu et al., 2009; Su et al., 2019; Venske et al., 2019; Ma et al., 2020). Most of those resistance QTLs originated from Asian germplasm such as the Chinese wheat cultivars ‘Sumai-3’ and ‘Wangshuibai’ or landraces (Bai et al., 1999; Anderson et al., 2001; Buerstmayr et al., 2009; Xue et al., 2010; Steiner et al., 2017) and wild relatives (*Fhb3*, *Fhb6*, and *Fhb7*; Qi et al., 2008; Cainong et al., 2015; Guo et al., 2015). Nevertheless, the transfer of resistance QTLs from wild relatives, landraces, or exotic materials is challenging due to linkage drag and adaptability issues. Thus, only a few QTLs with a major effect on FHB resistance, in particular *Fhb1*, have been successfully employed in wheat breeding programs (Bai et al., 2018). Contrarily, the majority of the germplasm from the hard winter wheat region of the US relies upon the variation in FHB resistance from native sources including cultivars ‘Everest,’ ‘Overland,’ ‘Lyman,’ and ‘Expedition’ (Clinesmith et al., 2019; Zhang et al., 2022). However, the identification of genomic regions underlying native resistance and the development of reliable markers are essential for pyramiding those QTLs to maintain an effective level of FHB resistance in locally adapted wheat backgrounds. Thus, it is important to determine the genetic basis of native FHB resistance in the germplasm from the HWW regions.

Though numerous QTLs for FHB resistance have been identified using traditional linkage mapping, this approach can encompass limited diversity. Genome-wide association study (GWAS) provides a good alternative by providing a much higher resolution to capture insights into the genetic architecture of complex traits because of historically accumulated mutations or recombination events (Scherer and Christensen, 2016; Sidhu et al., 2020). GWAS has been successfully used to dissect many traits of economic importance in wheat (Sukumaran et al., 2014; Sidhu et al., 2020; AlTameemi et al., 2021) including a several studies on FHB resistance (Kollers et al., 2013; Arruda et al., 2016; Wang et al., 2017; Tessmann et al., 2019; Larkin et al., 2020; Zhu et al., 2020). However, GWAS on FHB resistance has not been reported for the US hard winter wheat. Furthermore, recent developments in multi-locus GWAS (ML-GWAS) models have improved the power and reliability of this approach to identify causal loci for complex traits. For instance, more powerful methods like FarmCPU and BLINK have improved the ability of GWAS to detect the loci of smaller effects (Liu et al., 2016; Huang et al., 2019). Apart from these models, several important ML-GWAS models have been reported to outperform conventional single-locus GWAS models, which include the multi-locus random-SNP-effect mixed linear model (mrMLM), fast multi-locus random-SNP-effect mixed linear model (FASTmrMLM), fast multi-locus random-SNP-effect efficient mixed-model analysis (FASTmrEMMA), iterative modified-sure independence screening Expectation–Maximization-Bayesian least absolute shrinkage and selection operator (ISIS EM-BLASSO), polygenic-background-control based least angle regression plus empirical Bayes (pLARmEB), and pKWmEB (Wang et al., 2016; Tamba et al., 2017; Zhang et al., 2017; Ren et al., 2018; Wen et al., 2018). The ML-GWAS models are not only more reliable and efficient but also overcome the requirement of multiple testing correction that may result in false negatives (Zhang et al., 2019).

Majority of the GWAS studies make use of assembled diversity panels or landraces in various crop species (Ward et al., 2019). However, several studies effectively used the panels consisting of elite breeding lines to dissect the genetic basis of various traits of economic importance (Sukumaran et al., 2014, 2018). This approach permits identification, mapping, and the direct transfer and pyramiding of identified QTLs to the new backgrounds in the breeding programs without any linkage drag. Unfortunately, such studies have not been explored for FHB resistance in the US hard winter wheat breeding germplasm. Here we conducted such a study by genotyping a panel of elite lines from the South Dakota State University (SDSU) breeding program using genotyping-by-sequencing (GBS) and phenotyped for FHB resistance in a controlled FHB field nursery for two years to uncover the genetic basis of native FHB resistance. The specific objectives of this study were to (i) evaluate the genetic basis of FHB resistance in hard winter wheat elite breeding material; (ii) identify markers associated with the QTLs to facilitate marker-assisted selection; (iii) identify putative candidate genes in the QTL regions that were significantly associated with FHB resistance.

## Materials and Methods

### Plant Materials and FHB Evaluation

A set of 257 advanced and elite breeding lines from the SDSU winter wheat breeding program was evaluated for FHB resistance in a mist irrigated field nursery in the 2019 and 2020 winter wheat-growing seasons. Most of the evaluated breeding lines were at either F_4:7_ or F_4:8_ filial generations. Among these lines, 169 were screened in the 2019 nursery and 154 in the 2020 nursery, with an overlap of 58 lines between the two seasons. Owing to the missing genotypic data or inconsistent replications, eight lines from 2019 and one line from 2020 were removed, leaving 257 unique lines for downstream analysis.

The FHB nurseries were planted at Brookings, South Dakota (44.3114 °N, 96.7984 °W) in the fall of 2018 and 2019, respectively, using a randomized complete block design with 2 or 3 replicates for different sets of lines. ‘Lyman’ and ‘Emerson’ were used as resistant checks while ‘Flourish’ was used as the susceptible check in both experiments. Each experimental unit consisted of a 1-meter-long row plot with an inter-row spacing of 20 cm. The experimental plots were managed using the regional standard cultural practices. Days to heading (DTH) were recorded by calculating the Julian date when 50% of the plot had completely emerged heads. Plant height (PH) was measured from the soil surface to the top of spikes excluding awns at maturity.

The FHB nurseries were inoculated using corn kernel spawn and spraying a spore suspension of *F. graminearum* isolates (SD-FG1) as described in Halder et al. (2019). Briefly, the Fusarium-infected corn kernels (scabby corn inoculum) were scattered in the field at the boot stage (Feekes 10), followed by another round at the heading stage (Feekes 10.1) to ensure maximum infection in the FHB nursery. In addition, wheat plots were also inoculated by spraying a conidial suspension (100,000 spores/ml) at 50% anthesis to avoid any disease escape. The nursery was misted using an overhead mist irrigation system for two min with 15 min intervals from 19:00 to 7:00 h daily to maintain a humid micro-environment for disease infection. FHB disease incidence (INC) and severity (SEV) were scored for 20 spikes per genotype in each replication 21 days after anthesis using the scale described by Stack and McMullen (2011). The FHB disease index (DIS) was calculated as (INC × SEV)/100 (Stack and McMullen, 2011). The plots were harvested and threshed using a low air-speed thresher to prevent the loss of shriveled kernels. The harvested grain from each plot was visually scored for fusarium-damaged kernels percentage (FDK) by comparing each grain sample to a set of known FDK standards.^1^

### Statistical Analysis

The phenotypic data from two seasons was analyzed to obtain the best linear unbiased estimates (BLUEs) for FHB traits using the following model:


$$y_{ijk}=\mu +E_{i}+R_{j\left(i\right)}+G_{k}+{GE}_{ik}+e_{ijk}$$

where *y_ijk_* is the trait of interest, μ is the overall mean, *E*_i_ is the effect of the *i*^th^ environment, *R_j(i)_* is the effect of the *j*^th^ replicate nested within the *i*^th^ environment, *G_k_* is the effect of the *k*^th^ genotype, *GE_ik_* is the effect of the genotype × environment (G × E) interaction, and *e_ijk_* is the residual error term. The broad-sense heritability (H^2^) of a trait of interest in a combined environment analysis was assessed based on the variance estimates from the linear mixed model as follows:


$$H^{2}=\frac{\sigma_{g}^{2}}{\sigma_{g}^{2}+\sigma_{ge}^{2}/\mathrm{nLoc}+\sigma_{e}^{2}/\left(\mathrm{nLoc}\;x\;\mathrm{nRep}\right)}$$

where $\sigma_{g}^{2}$and $\sigma_{e}^{2}$, are the genotype and error variance components, $\sigma_{ge}^{2}$ is the G × E interaction variance component and *nLoc* is the number of environments in the analysis. The analysis was performed in META-R (Alvarado et al., 2019) which is based on the ‘lme4’ (Bates et al., 2015) R package. The summary statistics, correlations, visualization, and comparison tests were performed in R (R Core Team, 2021).

### Genotyping and Quality Control

The wheat panel was genotyped using the Genotyping-by-sequencing (GBS) procedure (Poland et al., 2012) at USDA Central Small Grain Genotyping Lab, Manhattan, KS. Briefly, the genomic DNA was extracted from leaf tissue at a three-leaf stage for each line using a cetyltrimethylammonium bromide (CTAB) method (Bai et al., 1999). GBS libraries were prepared by double restriction digestion with HF-*PstI* and *MspI* restriction enzymes (Poland et al., 2012) and sequenced in an Ion Proton sequencer (Thermo Fisher Scientific, Waltham, MA, United States). The Chinese Spring wheat genome reference RefSeq v2.0 (IWGSC, 2018; Zhu et al., 2021) was used to align the GBS reads with the default settings of Burrows-Wheeler Aligner v0.6.1.Single-nucleotide polymorphisms (SNPs) were called using the GBS v2.0 SNP discovery pipeline in TASSEL v5.0 (Bradbury et al., 2007). For quality control, unmapped SNPs and the SNPs with more than 30% missing values, minor allele frequency (MAF) of less than 5%, and more than 10% heterozygote frequency were filtered out. The remaining 9,321 high-quality SNPs were imputed using BEAGLE v4.1 (Browning and Browning, 2007) for further analysis.

### Population Structure and Linkage Disequilibrium Analysis

The principal component analysis (PCA) on the imputed SNP data of 257 lines was conducted to analyze the population structure using R (R Core Team, 2021) packages ‘SNPRelate’ (Zheng et al., 2012) and ‘ggplot2’ (Wickham, 2016). The population structure was also assessed using a Bayesian model-based clustering program, STRUCTURE v2.3.4 using an Admixture model (Pritchard et al., 2000) assuming 10 subgroups (*K = 1–10*) with 10 independent runs for each subgroup using a burn-in period of 10,000 iterations followed by 20,000 Monte Carlo iterations. The analysis was implemented in parallel using StrAuto v1.0 on the SDSU high-performance computing (HPC) cluster (Chhatre and Emerson, 2017; Tange, 2018). An *ad hoc* statistic (DeltaK) was used to infer the most likely number of subgroups using STRUCTURE HARVESTER (Evanno et al., 2005; Earl and vonHoldt, 2012). The linkage disequilibrium (LD) parameters (*r^2^*) for the whole genome, as well as each sub-genome, were estimated separately in TASSEL v5.0 (Bradbury et al., 2007) by computing *r^2^* values for all pairwise markers using a sliding window size of 50 markers. LD decay over genetic distance was visualized by fitting a nonlinear model using the modified Hill and Weir method (Hill and Weir, 1988) in R (R Core Team, 2021).

### Multi-Locus Genome-Wide Association Analysis

ML-GWAS was used to identify marker-trait associations (MTAs) using BLUEs for FHB traits obtained using the mixed model analysis and 9,321 high-quality SNPs. For association analysis, we compared a total of eight ML-GWAS models. Two models, FarmCPU (fixed and random model, circulating probability unification) and the BLINK (Bayesian-information and linkage-disequilibrium iteratively nested keyway), were implemented in genomic association and prediction integrated tool (GAPIT) v3.0 (Wang and Zhang, 2021) in the R environment. In addition, we used six recently developed ML-GWAS methods including mrMLM, FASTmrMLM, FASTmrEMMA, pLARmEB, ISIS EM-BLASSO, and pkWmEB. These six models were implemented in the R package ‘mrMLM v4.0.2’ (Zhang et al., 2020) using default parameters. The ML-GWAS models included the estimated kinship (K) and the first two principal components from PCA as covariates to account for relatedness and the population structure. Based on the comparison using quantile-quantile (QQ) plots for all the models, we decided to report the results generated from the FarmCPU model because it showed the best control of false positives and false negatives. Furthermore, we used a strict threshold based on false discovery rate correction (FDR adj. *p* value <0.05) for multiple testing. Though final results were reported from a single best model (FarmCPU), we used the results from the other seven ML-GWAS models to validate the FarmCPU MTAs as reliable, if they were also identified by other models. The Manhattan plots and QQ plots were generated using the R package ‘CMPlot’ to visualize the results from the FarmCPU analysis.

We also used a pairwise t-test to compare differences in trait means for different alleles of significant MTAs. For each MTA, mean trait values for two groups of alleles (resistant v/s susceptible) were compared using a t-test and visualized using boxplots with R package ‘ggplot2’ (Wickham, 2016). Furthermore, the allelic frequencies of significant MTAs were analyzed to compare the effect of the combination of resistance alleles for DIS and FDK. The 257 accessions were grouped based on the resistant alleles carried for each trait. These groups were compared using an FDR-adjusted pairwise t-test.

### Candidate Gene Analysis

Two highly significant MTAs for FDK were subjected to candidate gene analysis to identify genes with putative functions of interest. Linkage blocks harboring these two MTAs were deduced using the confidence interval method in Haploview (Barrett et al., 2005). These MTAs were physically mapped to Chinese Spring RefSeq v2.1 using marker sequences of significant SNPs (IWGSC, 2018; Zhu et al., 2021). The high-confidence (HC) genes from IWGSC v2.1 RefSeq annotation were extracted from a flanking window around each MTA based on the LD decay in the respective region. The HC genes were annotated manually using Blast2GO (Conesa et al., 2005) for the identification of genes of interest. Further, a gene expression browser^2^ and a thorough review of literature were used to exclude the unlikely candidates. For the gene expression browser, we used expression data from studies related to *Fusarium* infection in wheat (Borrill et al., 2016).

## Results

### Observed Variation for FHB Traits

**Figure 1:**
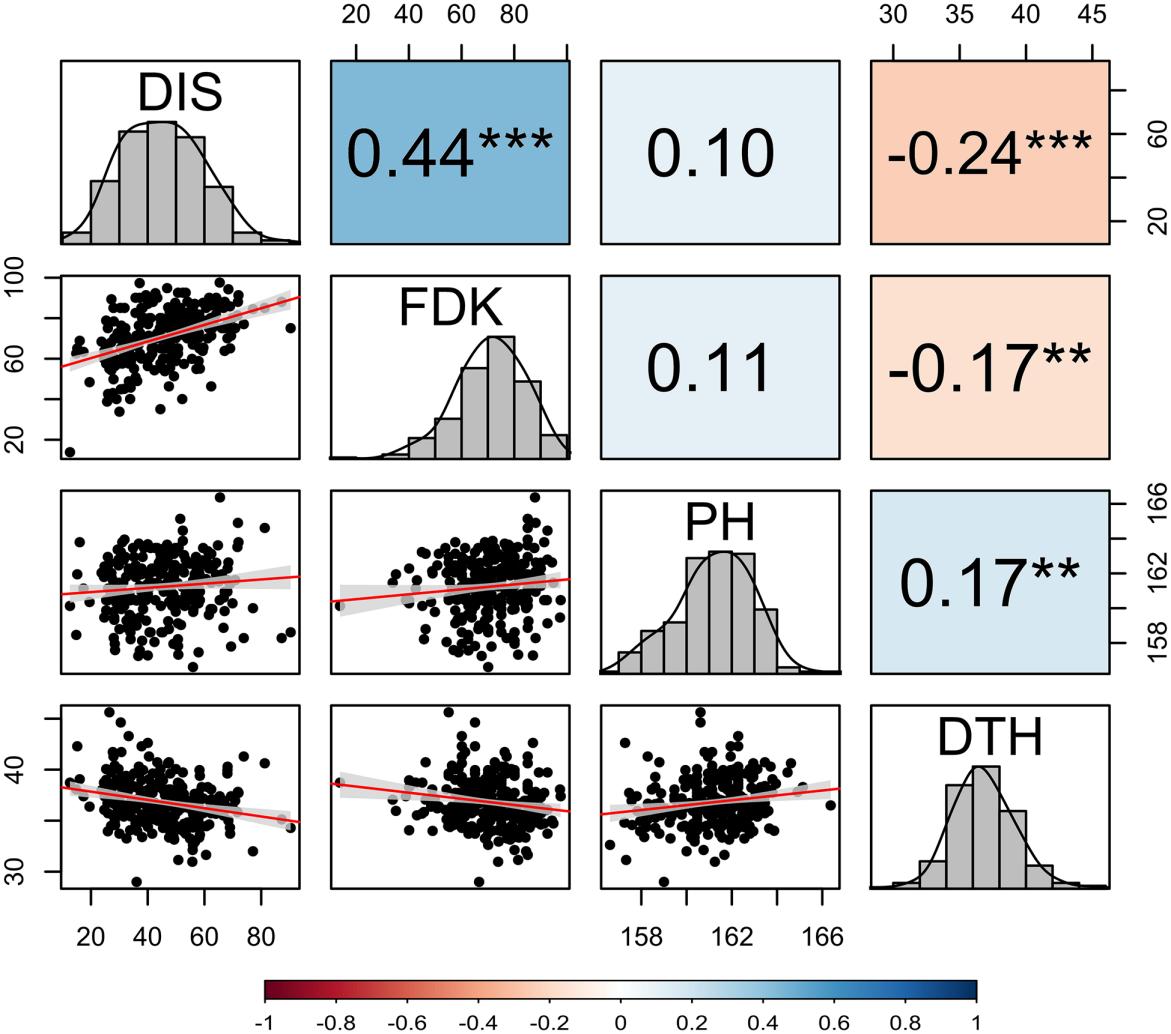
Correlation coefficients among investigated FHB traits, PH, and DTH calculated by using the best linear unbiased estimates (BLUEs) obtained from a combined analysis of hard winter wheat panel of 257 breeding lines over two years. DIS, FHB disease index; FDK, Fusarium-damaged kernel percentage; PH, plant height; and DTH, days to heading. The diagonal of the pair plot elucidates the frequency distribution for four traits and lower triangle shows the bivariate scatterplots with fitted lines. Statistically significant differences are denoted by an asterisk (^*^), where ^**^p ≤ 0.01 and ^***^p ≤ 0.001.

The BLUE values from two seasons exhibited a significant genotypic variation (*p* < 0.001) for DIS and FDK in the panel of 257 breeding lines. The variation for DIS ranged from 12.6 to 90.3, while the FDK ranged from 13.6 to 97.6 (Table 1). We observed a high broad-sense heritability (*H*^2^ = 0.85) for DIS, whereas a moderate heritability for FDK (0.76). The BLUE values for the disease indices (DIS) were 36.3 and 31.6, for the moderately resistant checks, Emerson and Lyman, respectively, whereas 77.1 for the susceptible check Flourish. Similarly, the FDK percentage was 48.9 and 31.5% for Emerson and Lyman whereas 84.5% for ‘Flourish.’ Pearson’s correlation coefficients between DIS and FDK estimated using the phenotypic BLUEs were significant (*r* = 0.44, *p* < 0.001; Figure 1). A significant negative correlation was observed between DIS and DTH (*r* = 0.24, *p* < 0.001) and between FDK and DTH (*r* = 0.17, *p* < 0.01). Further, DIS and FDK were not significantly correlated with PH (Figure 1).

**Table 1:** Phenotypic variation, variance estimates, and broad-sense heritability for studied FHB traits in the hard winter wheat panel of 257 breeding lines.

Trait	Genotypic variance^a^	Mean	Min	Max	CV^b^	H^2^
DIS^b^	109.1^***^	45.4	12.6	90.3	20.7	0.85
FDK^b^	156.2^***^	70.6	13.6	97.6	16.3	0.76

a Statistically significant differences are denoted by an asterisk (^*^), where

*** *p ≤ 0.001*.

b *DIS, FHB disease index; FDK, Fusarium-damaged kernel percentage; CV, coefficient of variation; H^2^, broad-sense heritability*.

### Genotyping, Population Structure, and Linkage Disequilibrium

**Figure 2:**
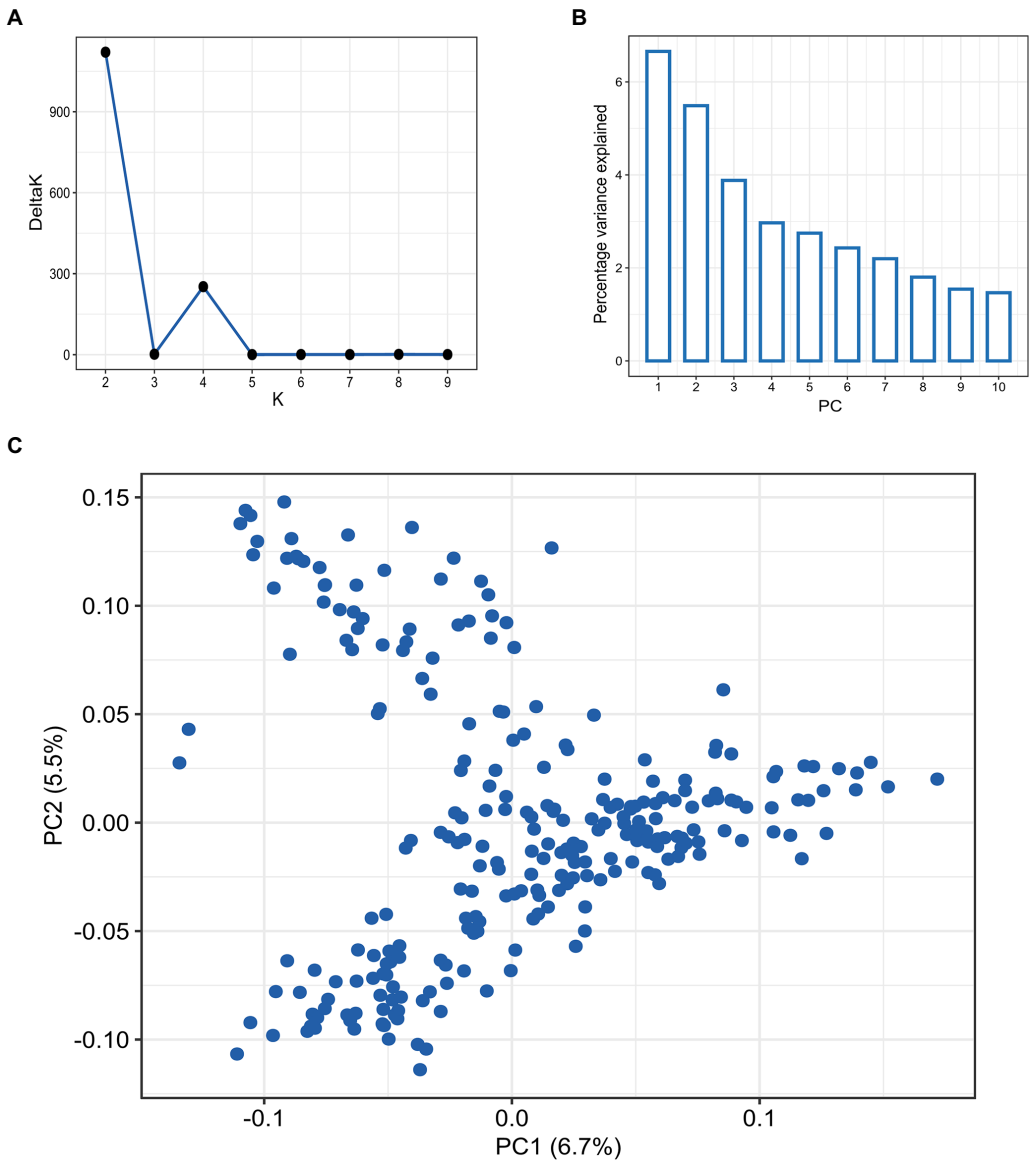
Population structure analysis in hard winter wheat breeding panel of 257 lines based on the 9,321 SNPs, **(A)** Evanno plot of Delta-K statistic from the STRUCTURE analysis. **(B)** Scree plot for first 10 components obtained from principal component analysis (PCA). **(C)** Scatterplot for the first two components (PC1 and PC2) from PCA.

Screening the panel using the GBS yielded 9,321 high-quality SNPs, with the B sub-genome having the highest SNP density (4,202; 45.1%) and the D sub-genome having the lowest SNP density (1,418; 15.2%; Supplementary Table S1). Among the 21 wheat chromosomes, 7A had the highest number of SNPs (796) whereas, chromosome 4D had the least (36 SNPs). The LD analysis revealed different patterns of LD decay among the three sub-genomes, with ~3.5 Mbp for the whole genome, and shorter LD decay distances for sub-genomes A and B than that for sub-genome D (Supplementary Figure S1). The population structure among 257 accessions was inferred using both PCA and STRUCTURE analysis (Figure 2). The DeltaK statistics based on STRUCTURE analysis revealed a major peak at *K* = 2, suggesting only two major groups in the panel (P1 and P2 for later reference; Figure 2A). The P1 comprised 138 accessions while the P2 consisted of 119 accessions. The subgroup P1 primarily consisted of breeding lines derived from the crosses involving parents from the southern HWW region of the US including breeding lines from Colorado, Kansas, Oklahoma, and Texas. Further, the P1 included important cultivars like Everest, Emerson, and Flourish, along with several breeding lines with Everest in its pedigree. In contrast, subgroup P2 was dominated by the cultivars from northern hard winter wheat region including Expedition, Ideal, Lyman, Overland, and Redfield, along with the breeding lines that have one these cultivars in their parentage. The results from PCA also showed considerable admixture in the population and suggested the presence of two subgroups in the panel in corroboration with STRUCTURE analysis (Figures 2B,C), with the first two principal components explaining around only 6.5 and 3.4% of the total variance, respectively.

### Genomic Loci Associated With FHB Traits

**Figure 3:**
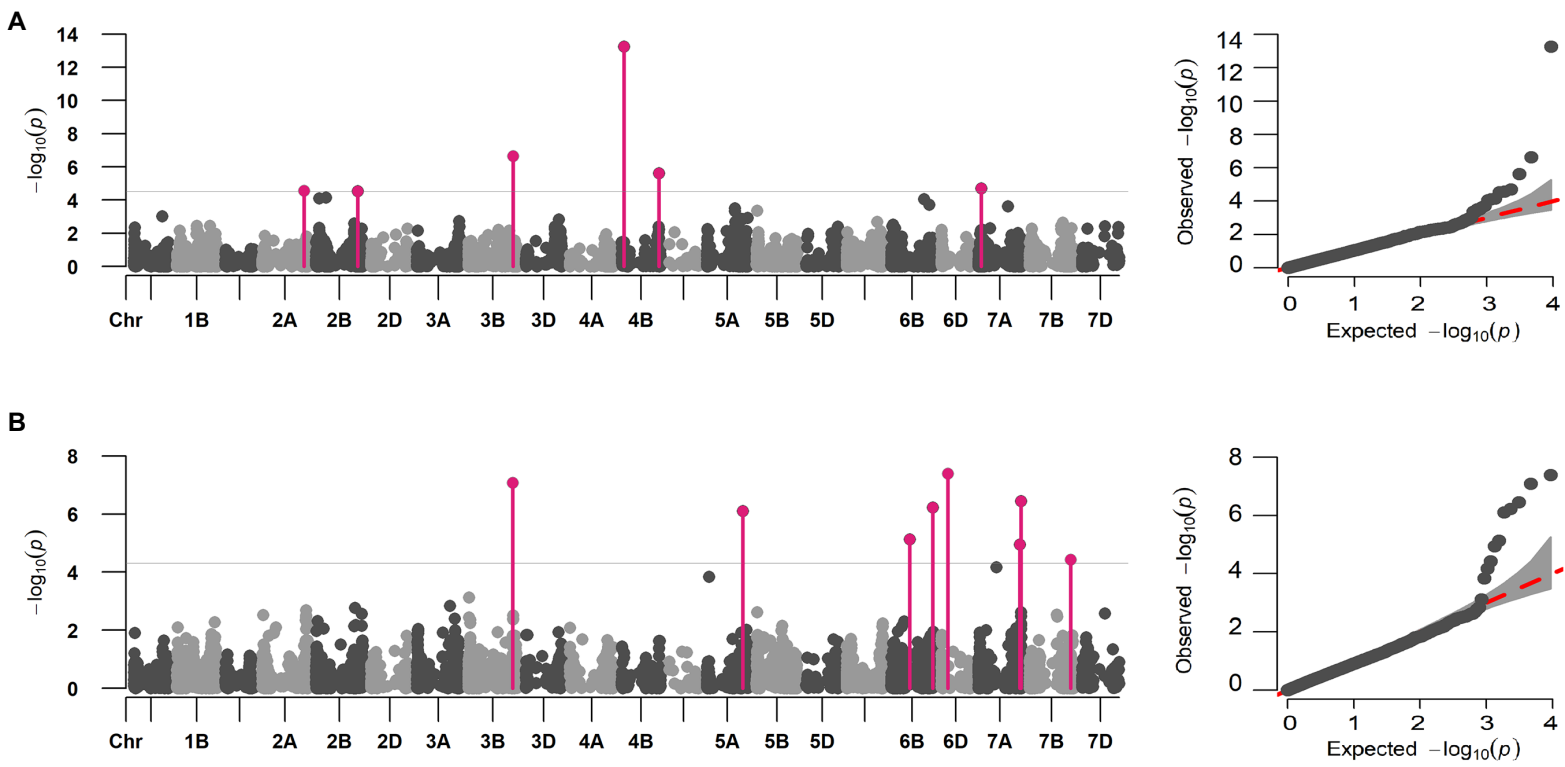
Multi-locus marker-trait association for DIS and FDK in hard winter wheat breeding panel using the FarmCPU model. Manhattan and QQ plot for **(A)** DIS and **(B)** FDK showing the distinct peaks for identified MTAs. The significant associations (FDR *p* < 0.05) are elucidated using solid pink vertical lines.

Initial GWAS using eight different ML-GWAS models identified a total of 52 marker-trait associations (MTAs) for DIS and 53 MTAs for FDK (Supplementary Table S2). Nevertheless, we compared the QQ plots generated from those models and found FarmCPU fit best for the two traits in terms of controlling false positives and false negatives (Supplementary Figure S2). Thus, the FarmCPU model was used to report final MTAs for DIS and FDK (Figure 3).

A total of six distinct MTAs on five chromosomes were significant for DIS using the FarmCPU model based on FDR corrected threshold (FDR adj. value of *p* < 0.05; Table 2; Figure 3A). Among them, five were considered more reliable as they were significant in at least one other ML-GWAS model except for one MTA on chromosome 2B (*S2B_725552556*; Table 2). The most significant MTA (*S4B_40315424*) was identified on the short arm of chromosome 4B that was physically mapped at 40 Mbp, followed by *S3B_773516625* at 773 Mbp on chromosome 3B. For FDK, a total of eight unique MTAs were identified on six different chromosomes using the FarmCPU model (Table 2; Figure 3B), with one each on chromosomes 3B, 5A, 6D, and 7B, two each on 6B and 7A, respectively. Among them, four MTAs on chromosomes 3B (*S3B_768314878*), 5A (*S5A_619020400*), 6B (*S6B_718194425*), and 7B (*S7B_707550430*) were reliable MTAs as those were identified by another ML-GWAS model (Table 2). Among the 14 MTAs for DIS and FDK, only two MTAs (*S3B_768314878* and *S4B_647586119*) were significant for both the traits (Supplementary Table S2).

**Table 2:** Significant marker-trait associations (MTAs) identified by genome-wide association studies (GWAS) using the FarmCPU model for FHB disease index (DIS) and Fusarium-damaged kernels (FDK) in the hard winter wheat panel of 257 breeding lines.

Trait	SNP	Chr	Pos^a^	MAF^b^	SNP effect	*p* value	FDR-adj *p* value	Another model^c^
DIS	S2A_722857568	2A	722,857,568	0.17	3.70	2.72E-05	0.046	1,2,3,6
	S2B_725552556	2B	725,552,556	0.18	3.98	2.94E-05	0.046	
	S3B_773516625	3B	773,516,625	0.14	−5.47	2.29E-07	0.001	1,2,3,6,7
	S4B_40315424	4B	40,315,424	0.16	−7.00	5.46E-14	0.000	1,2,3,4,5,6,7
	S4B_647586119	4B	647,586,119	0.26	3.51	2.37E-06	0.007	1,4,5
	S7A_48708273	7A	48,708,273	0.07	−4.74	1.96E-05	0.047	7
FDK	S3B_768314878	3B	768,314,878	0.40	3.61	8.23E-08	0.000	1,2,3,4
	S5A_619020400	5A	619,020,400	0.13	4.63	7.83E-07	0.000	1,2,7
	S6B_320696398	6B	320,696,398	0.12	4.05	7.41E-06	0.012	
	S6B_718194425	6B	718,194,425	0.23	3.64	5.98E-07	0.001	1,2,3,4,5,6,7
	S6D_110313864	6D	110,313,864	0.06	−7.50	4.09E-08	0.000	
	S7A_713432647	7A	713,432,647	0.12	−4.44	1.13E-05	0.015	
	S7A_738859192	7A	738,859,192	0.34	2.87	3.51E-07	0.001	
	S7B_707550430	7B	707,550,430	0.30	−2.86	3.7E-05	0.043	1,2,6

The MTAs were declared significant based on a false discovery rate (FDR) corrected *p* value threshold of 0.05.

a The physical position is based on IWGSC RefSeq v2.0 (IWGSC, 2018).

b MAF refers to minimum allele frequency for the corresponding MTA.

c This column enlists ML-GWAS model(s), which identified the corresponding MTA in addition to FarmCPU. The MTAs was referred to as ‘reliable’ if identified by at least two ML-GWAS models. Various ML-GWAS models are: 1, mrMLM; 2, FastmrMLM; 3, FastmrEMMA, 4, pLARmEB; 5, pKWmEB; 6, ISIS EM-BLASSO; and 7, BLINK.

**Figure 4:**
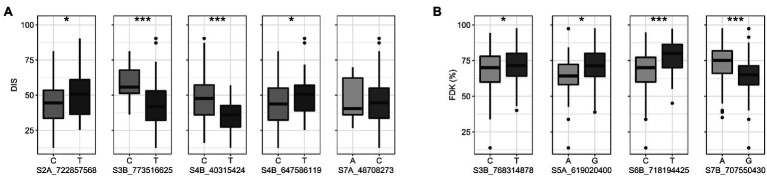
Boxplots showing the effect of two alleles (favorable v/s unfavorable) of the reliable MTAs (enlisted in Table 2) on the trait means for **(A)** DIS **(B)** FDK in the hard winter wheat panel. Statistically significant differences are denoted by an asterisk (^*^), where ^*^p ≤ 0.05 and ^***^p ≤ 0.001.

We compared trait means for DIS and FDK between the two alleles of the reliable MTAs (Figure 4). Of the five reliable MTAs for DIS, four exhibited a statistically significant difference in mean DIS scores between lines with the contrasting alleles (Figure 4A). The mean DIS score of the lines with resistance allele at *S3B_773516625* was 43.4%, significantly lower than those with susceptible allele (58.3%). Similarly, lines with a favorable allele at chromosome 4B (*S4B_40315424*) had a mean DIS score of 35.1% compared to 47.3% for lines carrying the susceptible allele. For FDK, all four reliable MTAs showed statistically significant differences in mean FDK percentage between lines carrying different alleles (Figure 4B). Intriguingly, favorable alleles at two MTAs (*S6B_718194425* and *S7B_707550430*) exhibited a decrease of around 13% of FDK over the unfavorable allele (Figure 4B).

### Additive Effect of Identified MTAs on FHB Resistance

**Figure 5:**
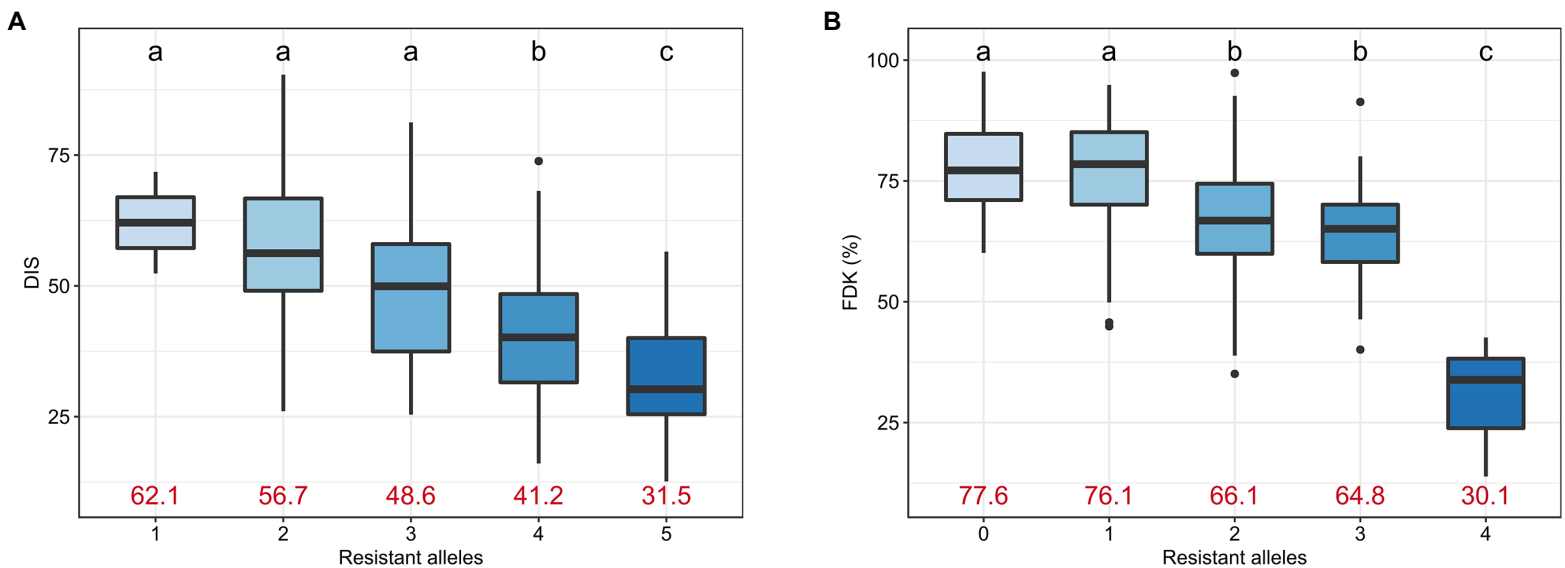
Boxplots comparing the trait performance of the hard winter wheat lines carrying different numbers of resistant alleles for **(A)** DIS and **(B)** FDK, compared using an FDR-adjusted Least Significance Difference (LSD) test. Different letters on top of the boxplots denote statistically different groups. The mean trait value for DIS and FDK for the corresponding group is given using red text.

We investigated the effect of accumulating favorable alleles at reliable MTAs on DIS and FDK. The panel of 257 accessions was categorized into groups based on the number of favorable alleles carried by accessions. For DIS, we observed five groups with lines carrying one, two, three, four, or all five resistance alleles at the associated loci, with no line carrying zero favorable alleles. We observed a significant decrease in the mean DIS score as the number of resistance alleles increased (Figure 5A). The mean DIS for the group of accessions having only ‘1’ resistance allele was 62.1, while the mean DIS for the group with all ‘5’ resistance alleles was 31.5 (Figure 5A). Similarly, the mean FDK was significantly reduced with an increase in the number of resistance alleles (Figure 5B).

### Relationship Between FHB and Height Genes

The most significant MTA for DIS (*S4B_40315424*) was identified at 40 Mbp on the short arm of chromosome 4B, where a semi-dwarfing gene *Rht1* is located. GWAS using the PH data of 257 accessions identified the most significant SNP at 40 Mbp (*S4B_40019966*) on chromosome 4B (Supplementary Table S3). Though the SNPs identified for DIS (*S4B_40315424*) and PH (*S4B_40019966*) were different, both the SNPs were 0.2 Mbp apart and in the same LD block, suggesting that this genomic region was associated with both traits in hard winter wheat. Further, we grouped the 257 accessions based on the allelic profile *S4B_40019966,* the MTA for PH in *Rht-B1* region. The two groups were compared using a t-test for differences in traits means for PH and DIS (Supplementary Figure S3). We observed significant differences (value of *p* < 0.001) in both PH and DIS among the two allelic groups. As expected, the allele associated with ‘tall’ phenotype had significantly lower DIS compared to the allele associated with the ‘dwarf’ phenotype (Supplementary Figure S3).

### Candidate Gene Analysis for Important Genomic Regions Associated With FDK

Candidate gene analysis was performed for two genomic regions (*S6B_718194425* and *S7B_707550430*) associated with FDK. The QTL associated with SNPs *S6B_718194425* and *S7B_707550430* were selected for candidate gene analysis as the LD blocks harboring these MTAs were identified to be smaller than 2.5 Mbp (Supplementary Figure S4). We identified a 1.7 Mbp LD block was identified harboring *S6B_718194425* (Supplementary Figure S4a). Similarly, a 2.3 Mbp long linkage block was identified for *S7B_707550430* (Supplementary Figure S4b). Based on Chinese Spring RefSeq v2.1, 28 and 20 high-confidence genes were retrieved from the 6B and 7B genomic regions, respectively (Supplementary Table S4). Further analysis using the wheat expression browser^3^ with *Fusarium* specific studies, and comparison with literature identified 17 genes with putative functions of interest (Table 3). The functional annotation showed that several of these genes encode putative proteins of interest including cytochrome P450 714C2-like, aquaporin PIP1-5-like, disease resistance protein RGA5-like, NBS-LRR disease resistance protein, hydroquinone glucosyltransferase-like and others (Table 3). Among the 17 genes, four (*TraesCS6B02G448800, TraesCS6B02G450000, TraesCS6B02G450200,* and *TraesCS6B02G450500*) from 6B and three genes (*TraesCS7B02G417000*, *TraesCS7B02G429800* and *TraesCS7B02G430000*) from 7B were of specific interest as they exhibited a differential expression between mock and *Fusarium* inoculated spikes in Chinese Spring (Supplementary Figures S5 and S6) and could be useful for further investigation of these QTL regions.

**Table 3:** Candidate genes identified for two QTLs for FDK, *S6B_718194425* and *S7B_707550430*, with putative functions of interest and their functional annotation.

Gene ID^a^	Previous ID^b^	Start position^c^	Annotation
TraesCS6B03G1247100	TraesCS6B02G448800	717,691,663	cytochrome P450 714C2-like
TraesCS6B03G1248900	TraesCS6B02G449400	718,124,729	nicotinate N-methyltransferase 1-like
TraesCS6B03G1249200	TraesCS6B02G449500	718,134,558	disease resistance protein RGA5-like
TraesCS6B03G1249300	TraesCS6B02G449600	718,142,603	disease resistance protein RGA5-like isoform X1
TraesCS6B03G1249800	TraesCS6B02G450000	718,403,799	aquaporin PIP1-5-like
TraesCS6B03G1250200	TraesCS6B02G450200	718,437,357	aquaporin PIP1-5-like
TraesCS6B03G1250700	TraesCS6B02G450500	718,634,434	50S ribosomal protein L9, chloroplastic
TraesCS6B03G1251200	TraesCS6B02G450700	718,948,307	acyl-CoA-binding domain-containing protein 4-like
TraesCS6B03G1252500	TraesCS6B02G451300	719,516,962	NAC domain-containing protein 78-like
TraesCS7B03G1160200	TraesCS7B02G417000	706,703,917	hypothetical protein CFC21_105377
TraesCS7B03G1160400	TraesCS7B02G417100	706,707,637	NBS-LRR disease resistance protein
TraesCS7B03G1161500	TraesCS7B02G417300	706,844,055	putative disease resistance protein RGA3
TraesCS7B03G1162000	TraesCS7B02G417400	706,905,895	probable LRR receptor-like serine/threonine-protein kinase
TraesCS7B03G1166000	TraesCS7B02G429700	708,194,926	hydroquinone glucosyltransferase-like
TraesCS7B03G1167100	TraesCS7B02G429800	708,341,181	uncharacterized methyltransferase At2g41040
TraesCS7B03G1167300	TraesCS7B02G430000	708,543,954	uncharacterized protein LOC119341039
TraesCS7B03G1167600	TraesCS7B02G430200	708,568,667	putative disease resistance RPP13-like protein 1 isoform X1

a Gene ID based on the IWGSC RefSeq Annotation v2.1 (IWGSC, 2018; Zhu et al., 2021).

b Previous IDs for respective genes to the IDs used in IWGSC RefSeq Annotation v1.1 (IWGSC, 2018).

c Physical position of start points for respective genes are based on IWGSC RefSeq v2.0 (IWGSC, 2018).

## Discussion

The utilization of host resistance to develop FHB-resistant wheat cultivars is the most economical and sustainable approach to manage FHB. This necessitates the continuous identification and validation of novel sources of FHB resistance and their utilization in breeding programs using marker-assisted selection. Thus, previous research efforts have resulted in the identification of several major and many minor QTLs for FHB resistance including *Fhb1*, and pyramiding them in various genetic backgrounds, particularly in spring wheat, has contributed significantly towards improved FHB resistance (Steiner et al., 2017; Bai et al., 2018; Ghimire et al., 2020). Nevertheless, using resistance genes from wild introgressions (*Fhb3*, *Fhb6*) or exotic sources, such as Sumai3, introduces the linkage drag or undesirable agronomic traits, making it difficult to incorporate these resistance genes into the US regional breeding programs. Thus, breeders still heavily rely on native sources for improving the FHB resistance in their breeding programs.

The majority of the HWW cultivars from the US Great Plains, including the SDSU winter wheat program, do not carry *Fhb1* likely due to yield drag, and ‘TAM 205’ is the only released HWW variety carrying *Fhb1* to date (Zhang et al., 2022). Fortunately, several HWW cultivars including Everest, Overland, Lyman, Heyne, Century, and Hondo that exhibit moderate FHB resistance but do not carry *Fhb1* have been released in the US HWW region (Jin et al., 2013; Bai et al., 2018; Clinesmith et al., 2019; Zhang et al., 2022), showing the importance of the native FHB resistance in the regional wheat breeding programs. Further, various studies have successfully identified QTLs for native resistance using cultivars such as ‘Art,’ Everest, and Lyman (Clinesmith et al., 2019; Hashimi, 2019). Thus, we used a set of advanced and elite breeding lines from the SDSU program with parentage from the US hard winter wheat region to identify genomic regions associated with FHB resistance, which could be readily employed in developing improved cultivars through marker-assisted breeding.

In this study, we evaluated a total of 257 breeding lines for FHB resistance in controlled disease nurseries over 2 years, with 58 lines being evaluated over both the years. The correlation for DIS evaluations from the two nurseries was 0.78, and the correlation among FDK evaluation over the years was 0.56. The significantly positive correlation among two nurseries, along with consistent performance of check cultivars suggests the reliability of phenotypic evaluation for GWAS. After evaluation of DIS and FDK in a panel of advanced breeding lines, we observed a significant variation for both the traits, with DIS from 12.6 to 90.3% and FDK from 13.6 to 97.6%. As majority of lines in the panel did not have *Fhb1* based on the parentage, the significant genotypic variation observed for the two traits was mainly contributed by native resistance genes. Furthermore, we observed a moderate to high heritability for DIS and FDK, which was in corroboration with several previous studies (Larkin et al., 2020; Xu et al., 2020; Zhu et al., 2020).

FarmCPU is an improved multi-locus model that eliminates the drawbacks of the conventional single-locus models by using associated markers as covariates to perform marker tests within a fixed-effect model. Later, this algorithm uses a separate random effect model for optimization of the association between tested markers and the trait (Liu et al., 2016). Several recent studies have reported better control of false positives and false negatives using FarmCPU compared to single-locus as well as other multi-locus models, as observed in our study. Hence, we employed FarmCPU for ML-GWAS that identified a total of six and eight genomic regions associated with DIS and FDK, respectively (Table 2). As previous studies used various types of marker systems to map FHB resistance and several are mapped to Chinese Spring RefSeq 1.0, it is difficult to precisely compare the MTAs from the current study (mapped using Chinese Spring RefSeq 2.0) with previously identified regions. Therefore, we identified the approximate physical locations of previous QTLs and MTAs from the current study on Chinese Spring RefSeq 1.0 to facilitate the comparison and validation of the different loci for FHB resistance (Supplementary Table S5; IWGSC, 2018; Zhao et al., 2019).

Out of the six MTAs identified for DIS (Table 2), four were found in the proximity to previously reported loci for different types of FHB resistance, including type III and type IV resistance. One MTA identified at 718 Mbp on the long arm of chromosome 2A in the current study could be the same one (~709 Mbp) identified in a US soft winter wheat line ‘VA00W-38’ (Liu et al., 2012) for DON (type III) resistance. Another MTA on chromosome 3B (*S3B_773516625*) for DIS was mapped to 753 Mbp on RefSeq v1.0, which overlaps with the genomic region (~753 Mbp) for FHB type III resistance (DON) from a Canadian spring wheat cultivar ‘AAC Tenacious,’ validating the importance of this region (Dhariwal et al., 2020). Recently, a GWAS using soft red winter wheat germplasm from the US also reported a QTL in similar 3B region associated with both type II and type III resistance (Ghimire et al., 2022). Further, the MTA identified on 7AS (*S7A_48708273*) was mapped in a close proximity to a QTL for type III resistance (~50 Mbp) in soft red winter wheat germplasm from southern US (Larkin et al., 2020). In addition to this, several QTLs have been reported in this 7A genomic region (~28–68 Mbp) for different types of FHB resistance in previous studies (Zhang et al., 2010; Jiang et al., 2020; Thambugala et al., 2020).

Several studies have reported a co-localization of QTLs for FHB resistance with semi-dwarf genes such as *Rht-B1* and *Rht-D1*, with dwarfing alleles at these loci related to FHB susceptibility (Miedaner and Voss, 2008; Srinivasachary Gosman et al., 2008; Liu et al., 2012; Dhariwal et al., 2020; Thambugala et al., 2020; Goddard et al., 2021). In the current study, we identified a strong MTA for DIS on the short arm of chromosome 4B (37 Mbp), which co-localized with the *Rht-B1* region. Intriguingly, we did not observe a strong association between PH and any of the FHB traits based on Pearson’s correlation (Figure 1). Further, we conducted a GWAS for PH using 257 accessions and identified the most significant SNP for PH at the same location as for DIS (37 Mbp; Supplementary Table S3), which co-localized with the location of *Rht-B1*. In corroboration with previous studies (Srinivasachary Gosman et al., 2008; Buerstmayr and Buerstmayr, 2016), our results suggest that the susceptibility associated with dwarfing allele of *Rht-B1* might be caused by a potential linkage of susceptible genes with the dwarfing genes. Thus, the identification of recombinants where the linkage between *Rht* genes and the FHB susceptible gene(s) is broken could be useful for the breeders. Except for these four MTAs for DIS, we did not find any previously reported QTL in the proximity of MTAs on chromosome 2BL (*S2B_725552556*) and 4BL (*S4B_647586119*). Thus, these two MTAs could represent novel QTLs for FHB resistance in hard winter wheat.

Among the eight MTAs identified for FDK (Table 2), six MTAs were found in genomic regions previously reported for harboring FHB resistance loci. The MTA *S3B_768314878* for FDK was about 5 Mbp away from MTA *S3B_773516625* that was identified for DIS in this study, suggesting a possible pleiotropic effect on both traits. As discussed earlier, previous studies also identified QTLs for different types of FHB resistance, including disease severity and DON at a similar position, suggesting this is an important region for FHB resistance on 3BL (Dhariwal et al., 2020; Larkin et al., 2020; Ghimire et al., 2022). Similarly, MTA *S5A_619020400* at 617 Mbp for FDK was found in the proximity of two previously reported QTLs, one for FDK mapped at ~596 Mbp in soft red winter wheat varieties AGS 2060 and AGS 2035 (Castro Aviles et al., 2020), and another for DON mapped at 621 Mbp in a Canadian spring wheat cultivar AAC Tenacious (Dhariwal et al., 2020). Further, two MTAs (*S6B_320696398* and *S6B_718194425*) identified for FDK were on the different arms of chromosome 6B at 314 Mbp and 708 Mbp, respectively (Table 2). The MTA (*S6B_718194425*) on 6BL aligned within the confidence interval of a meta-QTL for FHB resistance reported by Venske et al. (2019). However, we did not find any previously reported loci for FHB resistance around 314 Mbp on the 6BS. Another MTA (*S6D_110313864*) from this study was mapped on the short arm of 6D. Although a few studies reported QTLs for FHB resistance in this region, we were unable to compare their exact locations with *S6D_110313864* due to different marker systems used in these studies.

Two MTAs (*S7A_713432647* and *S7A_738859192*) for FDK identified in this study correspond to 707 Mbp and 731 Mbp on CS RefSeq v1.0 (Table 2). Several studies have reported QTLs for FHB resistance in the vicinity of these genomic regions. Among them, one QTL for FDK was mapped between 611 and 724 Mbp in the ‘Nanda 2419 × Wangshuibai’ population (Li et al., 2008). Additionally, a recent GWAS using soft red winter wheat germplasm reported a QTL for type III and type IV resistance that was mapped at 738 Mbp, which perfectly co-localizes with the MTA from current study (Gaire et al., 2021). Previously, Larkin et al. (2020) also reported a QTL for type II resistance at 709 Mbp on chromosome 7A, which is in close proximity to the MTA *S7A_713432647* from this study. Other QTLs have also been reported for different FHB traits in the 7A region but their physical positions are not available for comparison with the newly identified MTAs (Li et al., 2012; Lu et al., 2013). Further, a significant MTA was identified at 698 Mbp on chromosome 7B, which is close to the QTL for DON mapped at ~718 Mbp in soft red winter wheat (Castro Aviles et al., 2020) and a QTL for FHB resistance was also mapped at ~683 Mbp from a cross of Ningmai-9 × Yangmai-158 (Jiang et al., 2020). Two recent GWAS using soft red winter wheat germplasm from the US reported QTLs for type II resistance at around 716 Mbp (Larkin et al., 2020) and for DON at 723 Mbp on chromosome 7B (Gaire et al., 2021).

Overall, we identified 14 genomic regions associated with DIS and FDK in the current study. Out of these, 10 MTAs were co-localized with previously reported loci for different FHB traits, thus our study validates the previously reported QTLs in hard winter wheat germplasm with a higher mapping resolution. Further, the accessions harboring multiple FHB QTLs (Supplementary Table S6) could be directly employed in wheat breeding and the SNPs associated with these QTLs from the current study could be used to develop Kompetitive allele specific PCR (KASP) markers to effectively track and pyramid these reliable FHB QTLs in diverse backgrounds using marker-assisted selection (Gill et al., 2019). We also identified four putative novel QTLs associated with FHB resistance that could be subjected to further investigation.

Further, we performed candidate gene analysis for two important genomic regions associated with FDK (*S6B_718194425* and *S7B_707550430*) to identify genes with putative functions of interest that could be used for further investigation of regions harboring these QTLs. We selected these two regions for candidate gene analysis based on the generation of LD blocks harboring significant SNPs. The LD analysis showed that the linkage blocks harboring these two SNPs were smaller than 2.5 Mbp, which seems to be appropriate region for identifying putative candidate genes. In wheat, the majority of cloned disease resistance genes encode intracellular immune receptors of the nucleotide-binding-site–leucine-rich repeat (NBS-LRR) family, receptor-like kinases (RLKs), or wall-associated kinases (WAKs) as the protein product (Keller et al., 2018). However, two recently characterized FHB resistance genes, *Fhb1* and *Fhb7*, were reported to have different mechanisms. Several candidates for *Fhb1* have been reported including a pore-forming toxin-like (PFT) gene encoding a chimeric lectin with two agglutinin domains (Rawat et al., 2016); and a mutation of a histidine-rich calcium-binding protein gene (Bai et al., 2018; Li et al., 2019; Su et al., 2019). Nevertheless, none of these genes share any conserved domains related to the disease-resistance gene cloned in plants (Li et al., 2019; Su et al., 2019). Recently, a gene encoding a glutathione S-transferase (GST) was determined as the *Fhb7*, which can detoxify pathogen-produced toxins by conjugating a glutathione (GSH) unit onto the epoxide moieties of the pathogenic molecule (Wang et al., 2020). Based on this information, we were able to identify several putative candidate genes for two genomic regions (Table 3), including several genes encoding putative disease resistance proteins such as LRR receptor-like serine/threonine-protein kinase, or nicotinate N-methyltransferase 1-like proteins (Table 3), that may play a role in the process of intracellular detection of pathogen-derived molecules and signal transduction (Zhou et al., 1995). Further, we analyzed expression data from *Fusarium*-infected spikes using a wheat expression browser (Borrill et al., 2016) and identified several differentially expressed genes in the target 6B and 7B regions between mock-inoculated and *Fusarium* inoculated spikes (Supplementary Figures S5 and S6). These genes could be helpful in the further investigation of these genomic regions for their role in FHB resistance.

In summary, the current study provides new insights into the genetic basis of native FHB resistance in hard winter wheat germplasm from the US Great Plains region. The study validates the role of 10 genomic regions in FHB resistance in wheat including HWW germplasm, providing more confidence for the employment of these regions in breeding programs. Four putative novel QTLs and all reported SNP markers can facilitate the deployment of these QTLs through marker-assisted selection. Further, the information on genomic regions associated with FHB resistance could be useful for the breeders to improve the genomic selection models to select breeding lines with improved FHB resistance.

## Data Availability Statement

The datasets used for analysis in this study can be found in online repository. The names of the repository can be found in the article/Supplementary Material.

## Author Contributions

SS and JZ conceptualized the experiment and designed the methodology. JZ, NB, JH, and SA performed the investigation. HG and SS performed the data curation. HG and JZ performed the data analysis, visualization, and software implementation. AB, PA, and GB carried out genotyping and SNP discovery. JZ, HG, and SS wrote the original manuscript. GB, SA, and BT contributed to the interpretation of results. SS provided overall supervision. All authors contributed to manuscript revision and approved the final manuscript.

## Funding

This project was collectively funded by the USDA hatch projects SD00H695–20, USDA-ARS agreement 59–0206–0-177 (USDA-USWBSI), and the USDA Agriculture and Food Research Initiative Competitive grants 2022–68013-36439 (Wheat-CAP) from the USDA National Institute of Food and Agriculture and South Dakota Wheat Commission grant 3X1340.

## Conflict of Interest

The authors declare that the research was conducted in the absence of any commercial or financial relationships that could be construed as a potential conflict of interest.

## Publisher’s Note

All claims expressed in this article are solely those of the authors and do not necessarily represent those of their affiliated organizations, or those of the publisher, the editors and the reviewers. Any product that may be evaluated in this article, or claim that may be made by its manufacturer, is not guaranteed or endorsed by the publisher.

## Footnotes

^1^ https://agcrops.osu.edu/newsletter/corn-newsletter/2015-21/rating-fusarium-damaged-kernels-fdk-scabby-wheat

^2^ http://www.wheat-expression.com/

^3^ http://www.wheat-expression.com
