# Supplementary material for: Multi-Locus Genome-Wide Association Studies to Characterize Fusarium Head Blight (FHB) Resistance in Hard Winter Wheat
Source: Front Plant Sci. 2022 Jul 25;13:946700. doi: 10.3389/fpls.2022.946700 (PMC9359313; doi:10.3389/fpls.2022.946700)

Supplementary Material

Multi-locus genome-wide association studies to characterize Fusarium head blight (FHB) resistance in hard winter wheat

Jinfeng Zhang[1$], Harsimardeep S. Gill[1$], Jyotirmoy Halder[1], Navreet K Brar[1], Shaukat Ali[1], Amy Bernardo[2], Paul St Amand[2], Guihua Bai[2], Brent Turnipseed[1], Sunish Sehgal[1*]

The genomic datasets used in this study can be found online at https://github.com/SunishSehgal/.

Supplementary Table S1. The distribution of 9,321 SNPs across 21 wheat chromosomes in the panel of 257 accessions.

Sub-genome	Chromosome	Number of SNPs	% SNPs
A	1	532	
	2	447	
	3	518	
	4	391	
	5	508	
	6	509	
	7	796	
Subtotal A		3,701	39.71
B	1	588	
	2	689	
	3	749	
	4	221	
	5	617	
	6	743	
	7	595	
Subtotal B		4,202	45.08
D	1	202	
	2	357	
	3	276	
	4	36	
	5	191	
	6	162	
	7	194	
Subtotal D		1,418	15.21
Total (A, B, and D)		9,321	100

Supplementary Table S2. Summary of all the MTAs for DIS and FDK identified in hard winter panel of 257 lines using eight ML-GWAS models. For FarmCPU and BLINK, the threshold to declare an association as significant was FDR adjusted *P* < 0.05. For the other six models, the associations were declared significant based on LOD > 3.

Trait	Model	SNP	Chromosome	Position[a]
DIS	BLINK	S2B_173523267	2B	173523267
	BLINK	S3B_773516625	3B	773516625
	BLINK	S4B_40315424	4B	40315424
	BLINK	S5A_621373206	5A	621373206
	BLINK	S6B_658140124	6B	658140124
	BLINK	S7A_42614676	7A	42614676
	BLINK	S7A_510433772	7A	510433772
	FASTmrEMMA	S2A_722857568	2A	722857568
	FASTmrEMMA	S3B_773516625	3B	773516625
	FASTmrEMMA	S4B_40315424	4B	40315424
	FASTmrEMMA	S7A_27378888	7A	27378888
	FASTmrMLM	S2A_722857568	2A	722857568
	FASTmrMLM	S2B_662259821	2B	662259821
	FASTmrMLM	S3B_773516625	3B	773516625
	FASTmrMLM	S4B_40315424	4B	40315424
	FASTmrMLM	S7A_27378888	7A	27378888
	FarmCPU	S2A_722857568	2A	722857568
	FarmCPU	S2B_725552556	2B	725552556
	FarmCPU	S3B_773516625	3B	773516625
	FarmCPU	S4B_40315424	4B	40315424
	FarmCPU	S4B_647586119	4B	647586119
	FarmCPU	S7A_48708273	7A	48708273
	ISIS EM-BLASSO	S2A_722857568	2A	722857568
	ISIS EM-BLASSO	S2B_662259821	2B	662259821
	ISIS EM-BLASSO	S3B_773516625	3B	773516625
	ISIS EM-BLASSO	S4B_40315424	4B	40315424
	ISIS EM-BLASSO	S5D_551464583	5D	551464583

	ISIS EM-BLASSO	S6B_96579342	6B	96579342
	ISIS EM-BLASSO	S7A_27378888	7A	27378888
	mrMLM	S2A_722857568	2A	722857568
	mrMLM	S2B_662259821	2B	662259821
	mrMLM	S3B_773516625	3B	773516625
	mrMLM	S4B_40315424	4B	40315424
	mrMLM	S6B_96579342	6B	96579342
	mrMLM	S7A_27378888	7A	27378888
	mrMLM	S7A_3972877	7A	3972877
	pKWmEB	S2B_789008561	2B	789008561
	pKWmEB	S4B_40315424	4B	40315424
	pKWmEB	S4B_642604572	4B	642604572
	pKWmEB	S6B_663525758	6B	663525758
	pKWmEB	S6D_70342	6D	70342
	pKWmEB	S7A_27378888	7A	27378888
	pKWmEB	S7A_510433772	7A	510433772
	pLARmEB	S2B_662259821	2B	662259821
	pLARmEB	S2B_789008561	2B	789008561
	pLARmEB	S4B_40315424	4B	40315424
	pLARmEB	S4B_642604572	4B	642604572
	pLARmEB	S5D_551464583	5D	551464583
	pLARmEB	S6B_634018818	6B	634018818
	pLARmEB	S6B_663525758	6B	663525758
	pLARmEB	S7A_27378888	7A	27378888
	pLARmEB	S7A_510433772	7A	510433772
FDK	BLINK	S5A_39854554	5A	39854554
	BLINK	S5A_619020400	5A	619020400
	BLINK	S6B_718194425	6B	718194425
	FASTmrEMMA	S2A_748396092	2A	748396092
	FASTmrEMMA	S3A_568391268	3A	568391268
	FASTmrEMMA	S3B_771956508	3B	771956508
	FASTmrEMMA	S6B_716336898	6B	716336898
	FASTmrMLM	S2A_748396092	2A	748396092

FASTmrMLM	S3A_12668487	3A	12668487
FASTmrMLM	S3A_568391268	3A	568391268
FASTmrMLM	S3B_771956508	3B	771956508
FASTmrMLM	S5A_618308582	5A	618308582
FASTmrMLM	S6B_716336898	6B	716336898
FASTmrMLM	S7B_707550430	7B	707550430
FarmCPU	S3B_768314878	3B	768314878
FarmCPU	S5A_619020400	5A	619020400
FarmCPU	S6B_320696398	6B	320696398
FarmCPU	S6B_718194425	6B	718194425
FarmCPU	S6D_110313864	6D	110313864
FarmCPU	S7A_713432647	7A	713432647
FarmCPU	S7A_738859192	7A	738859192
FarmCPU	S7B_707550430	7B	707550430
ISIS EM-BLASSO	S2A_748396092	2A	748396092
ISIS EM-BLASSO	S3A_530215377	3A	530215377
ISIS EM-BLASSO	S5A_39854554	5A	39854554
ISIS EM-BLASSO	S6B_716336898	6B	716336898
ISIS EM-BLASSO	S7B_472484704	7B	472484704
ISIS EM-BLASSO	S7B_707550430	7B	707550430
mrMLM	S2A_748396092	2A	748396092
mrMLM	S3A_12668487	3A	12668487
mrMLM	S3A_568391268	3A	568391268
mrMLM	S3B_771956508	3B	771956508
mrMLM	S5A_618308582	5A	618308582
mrMLM	S6B_716336898	6B	716336898
mrMLM	S7B_707550430	7B	707550430
pKWmEB	S2A_748396092	2A	748396092
pKWmEB	S3A_13811558	3A	13811558
pKWmEB	S3A_528989206	3A	528989206
pKWmEB	S5A_39854554	5A	39854554
pKWmEB	S6B_716171099	6B	716171099
pKWmEB	S7B_472484704	7B	472484704

pLARmEB	S1A_10439185	1A	10439185
pLARmEB	S1B_595397803	1B	595397803
pLARmEB	S2A_748396092	2A	748396092
pLARmEB	S2A_781672337	2A	781672337
pLARmEB	S2B_662259821	2B	662259821
pLARmEB	S3A_568391268	3A	568391268
pLARmEB	S3B_621303861	3B	621303861
pLARmEB	S3B_771956508	3B	771956508
pLARmEB	S4B_642901115	4B	642901115
pLARmEB	S5A_11637897	5A	11637897
pLARmEB	S6B_716336898	6B	716336898
pLARmEB	S7A_50507497	7A	50507497

[a]Physical position is based on IWGSC RefSeq v2.0 (IWGSC, 2018)

Supplementary Table S3. Table summarizing the GWAS results for plant height (PH) in hard winter wheat panel of 257 lines using the FarmCPU model. The threshold used to declare an association as significant was FDR adjusted *P* < 0.05.

SNP	Chromosome	Position[a]	*p*-value	MAF	FDR adjusted *p*-value
S2A_614445772	2A	614,445,772	4.8E-06	0.15	1E-02
S4B_40019966	4B	40,019,966	8.76E-13	0.18	8E-09
S5B_295150730	5B	295,150,730	1.23E-06	0.46	6E-03
S7B_9558646	7B	9,558,646	2.21E-06	0.38	7E-03

[a]Physical position is based on IWGSC RefSeq v2.0 (IWGSC, 2018)

Supplementary Table S4. Table enlisting all high-confidence genes identified in the two genomic regions (*S6B_718194425* and *S7B_707550430*) associated with FDK along with their annotation.

**Gene ID[a]**	**Previous Gene ID[b]**	**Start[c]**	**Annotation**
TraesCS6B03G1247100	*TraesCS6B02G448800*	717691663	cytochrome P450 714C2-like
TraesCS6B03G1247900	*TraesCS6B02G448900*	717933372	zinc finger protein ZAT5-like
TraesCS6B03G1248300	*TraesCS6B02G449000*	718038855	serine/threonine-protein phosphatase PP1-like
TraesCS6B03G1248400	*TraesCS6B02G449100*	718072499	hypothetical protein CFC21_090362
TraesCS6B03G1248500	*TraesCS6B02G449200*	718081314	rop guanine nucleotide exchange factor 3-like
TraesCS6B03G1248600	*TraesCS6B02G449300*	718084520	FHA domain-containing protein DDL-like
TraesCS6B03G1248900	*TraesCS6B02G449400*	718124729	nicotinate N-methyltransferase 1-like
TraesCS6B03G1249200	*TraesCS6B02G449500*	718134558	disease resistance protein RGA5-like
TraesCS6B03G1249300	*TraesCS6B02G449600*	718142603	disease resistance protein RGA5-like isoform X1
TraesCS6B03G1249400	*TraesCS6B02G449700*	718193470	NEP1-interacting protein-like 1
TraesCS6B03G1249500	*TraesCS6B02G449800*	718194677	predicted protein
TraesCS6B03G1249600	*TraesCS6B02G449900*	718351170	lysine-specific histone demethylase 1 homolog 3-like
TraesCS6B03G1249800	*TraesCS6B02G450000*	718403799	aquaporin PIP1-5-like
TraesCS6B03G1249900	*TraesCS6B02G450100*	718408744	laccase-15-like
TraesCS6B03G1250200	*TraesCS6B02G450200*	718437357	aquaporin PIP1-5-like
TraesCS6B03G1250500	*TraesCS6B02G450300*	718537206	peptidyl-prolyl cis-trans isomerase-like
TraesCS6B03G1250600	*TraesCS6B02G450400*	718550171	peptidyl-prolyl cis-trans isomerase-like
TraesCS6B03G1250700	*TraesCS6B02G450500*	718634434	50S ribosomal protein L9, chloroplastic
TraesCS6B03G1251100	*TraesCS6B02G450600*	718946263	4-coumarate--CoA ligase-like 3
TraesCS6B03G1251200	*TraesCS6B02G450700*	718948307	acyl-CoA-binding domain-containing protein 4-like
TraesCS6B03G1251400	*TraesCS6B02G450800*	718975754	hypothetical protein CFC21_090380
TraesCS6B03G1251500	*TraesCS6B02G450900*	718995041	hypothetical protein CFC21_090381
TraesCS6B03G1251800	*TraesCS6B02G451000*	719407030	csAtPR5, putative, expressed
TraesCS6B03G1251900	*TraesCS6B02G451100*	719416172	signal peptide peptidase-like 4
TraesCS6B03G1252300	*TraesCS6B02G451200*	719509971	phosphatidylinositol 4-phosphate 5-kinase 9-like
TraesCS6B03G1252500	*TraesCS6B02G451300*	719516962	NAC domain-containing protein 78-like

TraesCS6B03G1252600	*TraesCS6B02G451400*	719562411	probable mitochondrial saccharopine dehydrogenase-like oxidoreductase At5g39410
TraesCS6B03G1252700	*TraesCS6B02G451500*	719564065	predicted protein
TraesCS7B03G1159300	*TraesCS7B02G416700*	706566583	uncharacterized protein LOC123161760
TraesCS7B03G1159800	*TraesCS7B02G416800*	706617114	hydroquinone glucosyltransferase-like
TraesCS7B03G1160000	*TraesCS7B02G416900*	706684670	hypothetical protein CFC21_105376
TraesCS7B03G1160200	*TraesCS7B02G417000*	706703917	hypothetical protein CFC21_105377
TraesCS7B03G1160400	*TraesCS7B02G417100*	706707637	NBS-LRR disease resistance protein
TraesCS7B03G1161400	*TraesCS7B02G417200*	706838899	hypothetical protein CFC21_105379
TraesCS7B03G1161500	*TraesCS7B02G417300*	706844055	putative disease resistance protein RGA3
TraesCS7B03G1162000	*TraesCS7B02G417400*	706905895	probable LRR receptor-like serine/threonine-protein kinase At3g47570
TraesCS7B03G1163500	*TraesCS7B02G429100*	707818550	NADH-ubiquinone oxidoreductase chain 1
TraesCS7B03G1163700	*TraesCS7B02G429200*	707819633	ribosomal protein S13
TraesCS7B03G1164100	*TraesCS7B02G429300*	707825040	ribosomal protein L16
TraesCS7B03G1164800	*TraesCS7B02G429400*	707834318	cytochrome c biogenesis protein ccmFC
TraesCS7B03G1165100	*TraesCS7B02G429500*	707836552	Cytochrome c biogenesis Fc
TraesCS7B03G1165200	*TraesCS7B02G429600*	707836902	39 kDa protein in mitochondrial S-1 and S-2 DNA
TraesCS7B03G1166000	*TraesCS7B02G429700*	708194926	hydroquinone glucosyltransferase-like
TraesCS7B03G1167100	*TraesCS7B02G429800*	708341181	uncharacterized methyltransferase At2g41040,
TraesCS7B03G1167200	*TraesCS7B02G429900*	708370570	AAA-ATPase ASD, mitochondrial-like
TraesCS7B03G1167300	*TraesCS7B02G430000*	708543954	uncharacterized protein LOC119341039
TraesCS7B03G1167400	*TraesCS7B02G430100*	708558030	uncharacterized protein LOC123162851
TraesCS7B03G1167600	*TraesCS7B02G430200*	708568667	putative disease resistance RPP13-like protein 1 isoform X1

[a]Gene ID based on the IWGSC RefSeq Annotation v2.1 (IWGSC 2018; Zhu et al. 2021)
[b]Previous IDs for respective genes to the IDs used in IWGSC RefSeq Annotation v1.1 (IWGSC 2018)
[c]Physical position of start points for respective genes are based on IWGSC RefSeq v2.0 (IWGSC 2018)

Supplementary Table S5. The physical position of the significant SNPs associated with DIS and FDK corresponding to the Chinese Spring RefSeq v2.0 and RefSeq v1.0 (IWGSC, 2018).

SNP	RefSeq v2.0	RefSeq v1.0
S2A_722857568	722,857,568	718,979,885
S2B_725552556	725,552,556	717,127,511
S3B_773516625	773516625	758,202,918
S4B_40315424	40,315,424	37,575,130
S4B_647586119	647,586,119	648,502,365
S7A_48708273	48,708,273	46,257,001
S3B_768314878	768,314,878	752,885,972
S5A_619020400	619,020,400	617,251,477
S6B_320696398	320,696,398	314,551,477
S6B_718194425	718,194,425	708,786,003
S6D_110313864	110,313,864	88,831,444
S7A_713432647	713,432,647	707,834,631
S7A_738859192	738,859,192	731,888,363
S7B_707550430	707,550,430	698,229,993

Supplementary Table S6. List of lines carrying combination of favorable alleles for identified MTAs along with DIS and FDK scores.

Trait	ID	Pedigree	Favorable alleles	Trait score
DIS	SD19019-3	SD09118/Everest	5	12.60
	SD18272-1	CO06424/SD05266-1W-3//SyWolf	5	25.37
	SD18018-2	OK06840WRESL/Wendy//SD05W030/3/SD07W083-7	5	25.70
	SD15025-1	Radiant/SD07204//SD07165	5	27.21
	SD18333-7	SD07W083-4/KS031027NT-11	5	27.70
	SD18333-3	SD07W083-4/KS031027NT-11	5	29.77
FDK	SD19019-3	SD09118/Everest	4	13.85
	SD18094-4	SD09034/Everest	4	33.85
	SD19017-6	SD09118/SyWolf	4	42.60

Supplementary Figure S1. Intra-chromosomal linkage disequilibrium (LD) in the panel of 257 lines.

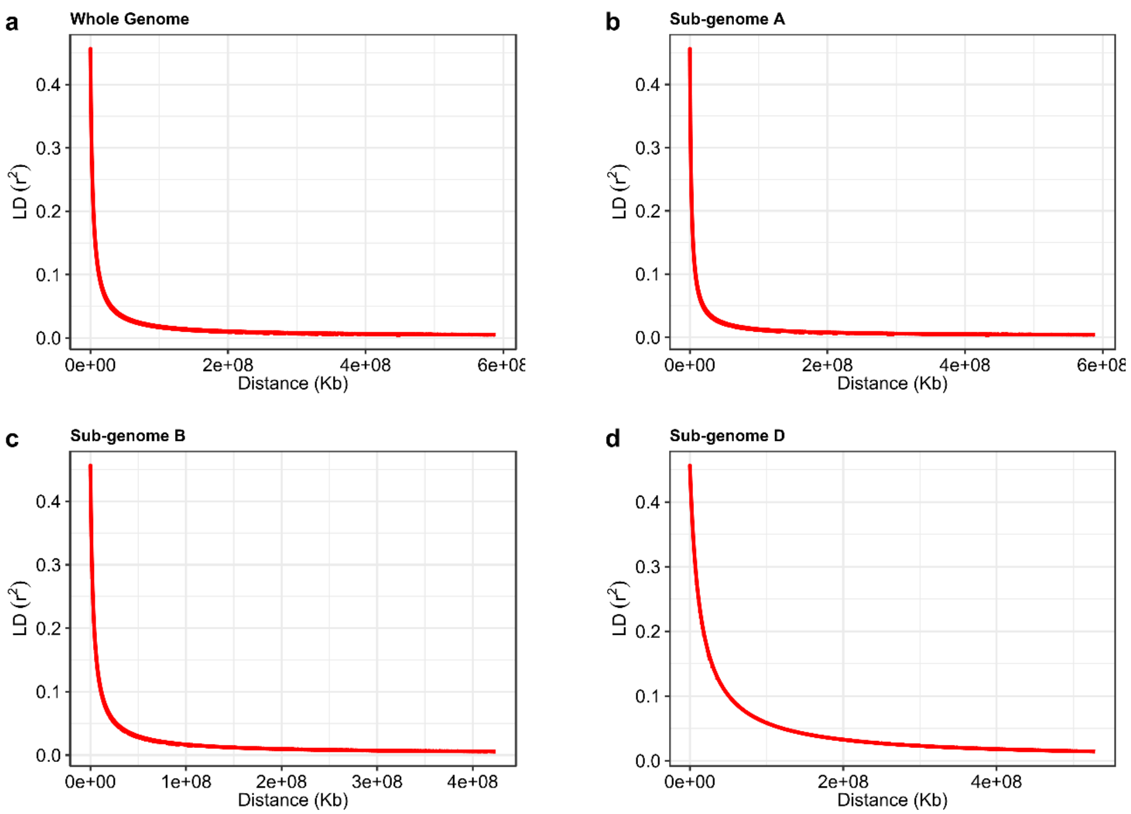

Supplementary Figure S2. QQ plots comparing the model performance of various ML-GWAS traits. The upper panel compares different ML-GWAS models for DIS, where (a) QQ plot using FarmCPU for DIS, (b) QQ plot using BLINK for DIS, and (c) a combined QQ plot for DIS obtained from six ML-GWAS models including mrMLM, FastmrMLM, FastmrEMMA, pLARmEB, pKWmEB, and ISIS EM-BLASSO. The lower panel shows the comparison of QQ plots for FDK, where (d) QQ plot using FarmCPU for FDK, (e) QQ plot using BLINK for FDK, and (f) a combined QQ plot for FDK obtained from six ML-GWAS models including mrMLM, FastmrMLM, FastmrEMMA, pLARmEB, pKWmEB, and ISIS EM-BLASSO.

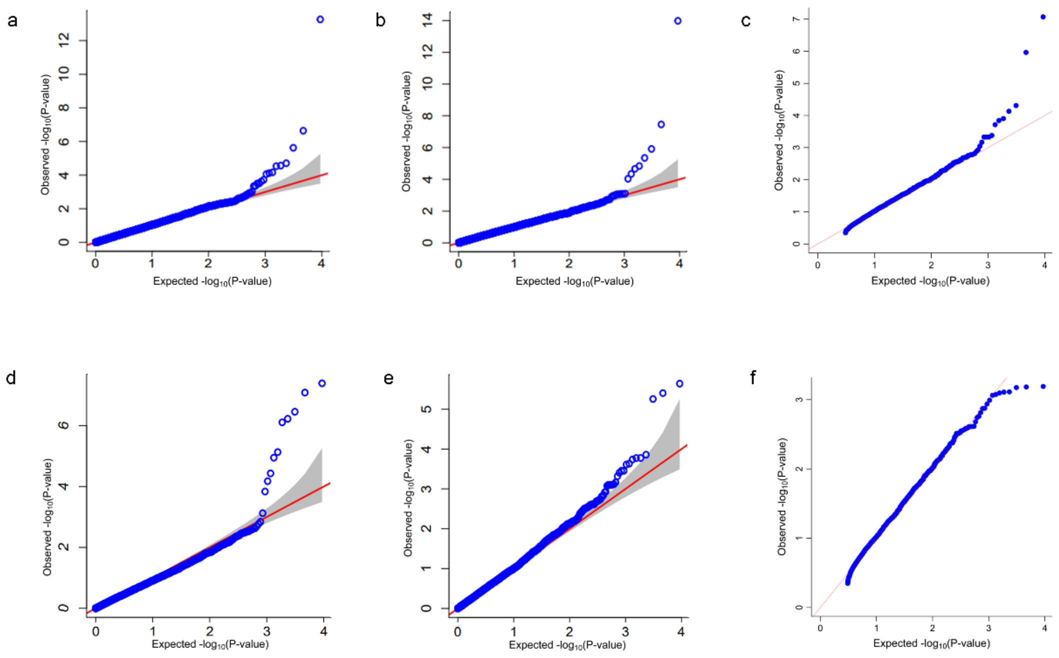

Supplementary Figure S3. Pairwise comparison among two groups based on allelic constitution of the MTA (*S4B*_40019966) for plant height in *Rht-B1* region. Boxplots elucidate the differences between the two allelic groups for (a) plant height, and (b) FHB disease index (DIS).

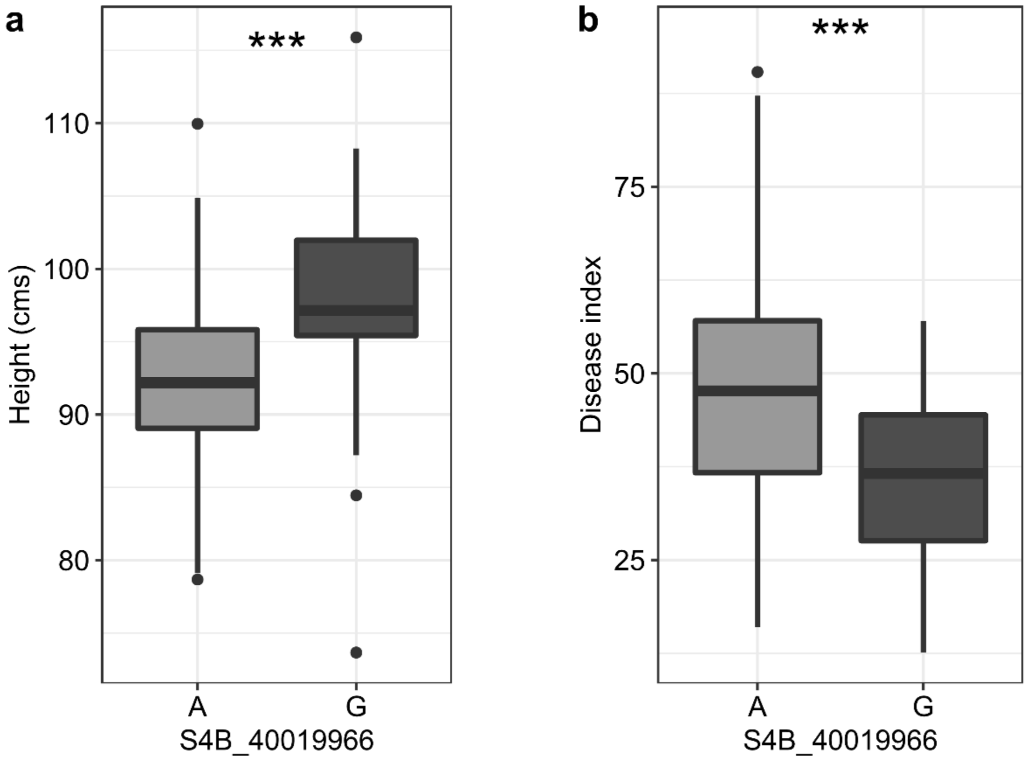

Supplementary Figure S4. Local linkage disequilibrium (LD) block for region harboring MTAs for FDK, (a) *S6B_718194425* and (b) *S7B_707550430*.

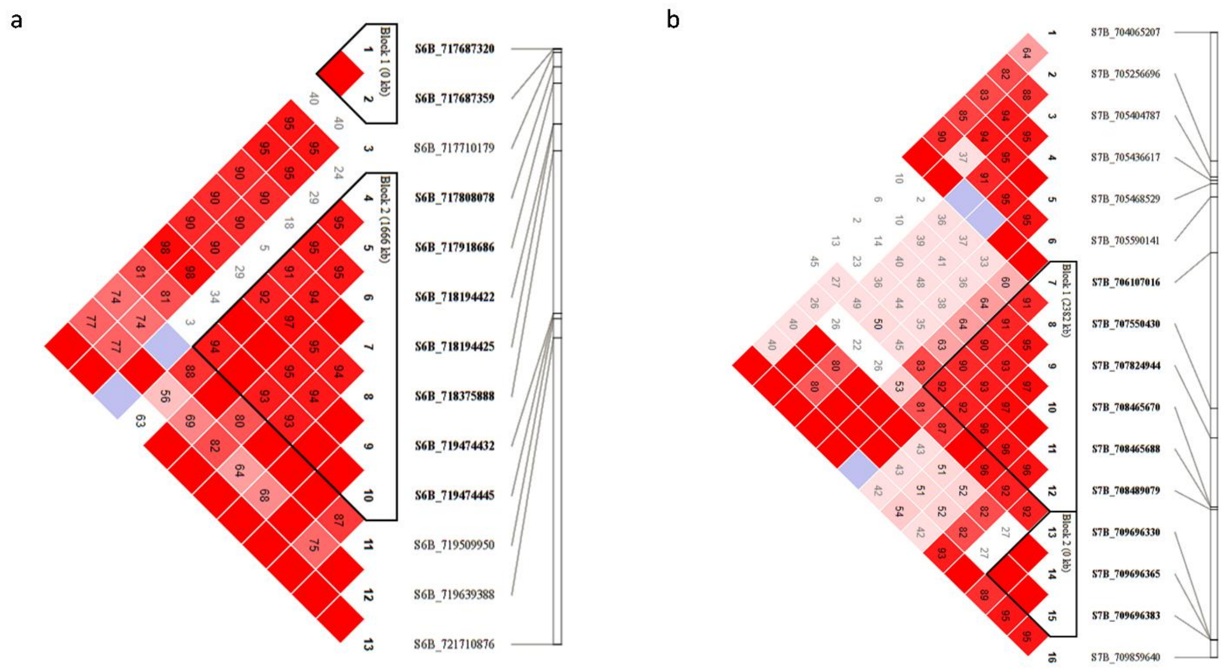

Supplementary Figure S5. Gene expression analysis for high confidence genes in the flanking region of SNP *S6B_718194425* across several studies for time courses of *Fusarium* infection. Gene expression is presented as a heatmap with Gene IDs based on IWGSC RefSeq v1.1 are listed on the top and the stages/tissues of expression on the side.

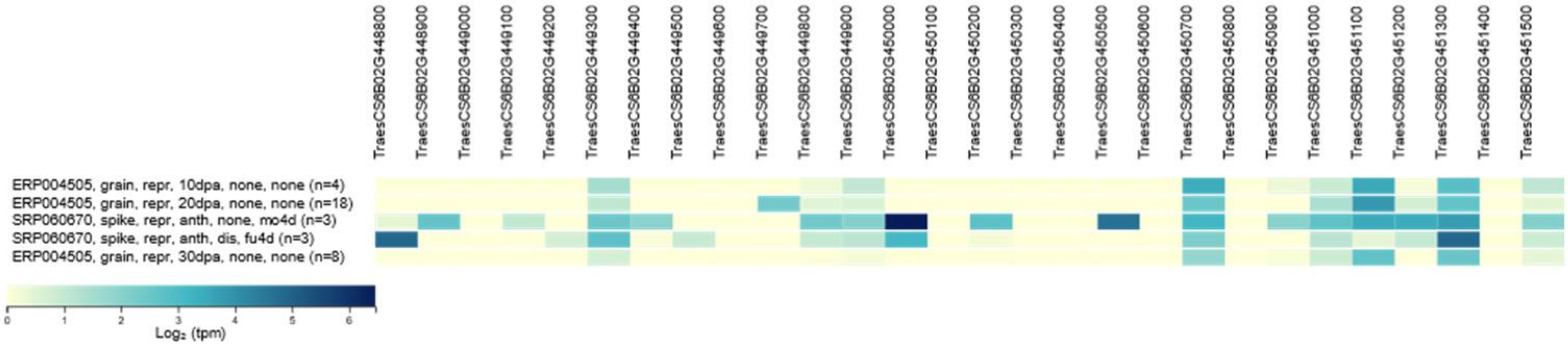

Supplementary Figure S6. Gene expression analysis for high confidence genes in the flanking region of SNP *S7B_707550430* across several studies for time courses of *Fusarium* infection. Gene expression is presented as a heatmap with Gene IDs based on IWGSC RefSeq v1.1 are listed on the top and the stages/tissues of expression on the side.

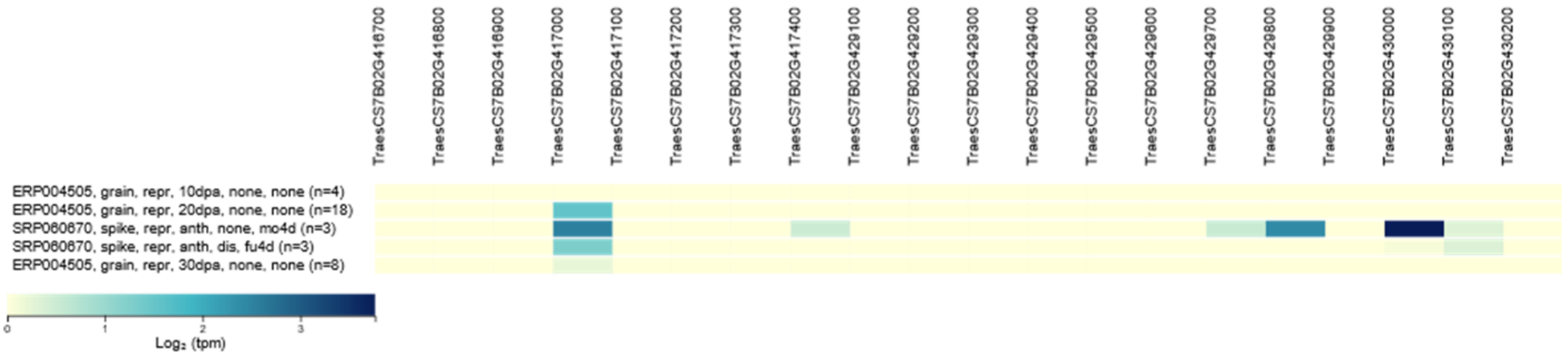

Supplement: Supplementary file 1 [file Data_Sheet_1.pdf]
